# Active Engagement, Protective Buffering, and Depressive Symptoms in Young-Midlife Couples Surviving Cancer: The Roles of Age and Sex

**DOI:** 10.3389/fpsyg.2022.816626

**Published:** 2022-02-17

**Authors:** Karen S. Lyons, Jessica R. Gorman, Brandon S. Larkin, Grace Duncan, Brandon Hayes-Lattin

**Affiliations:** ^1^Connell School of Nursing, Boston College, Chestnut Hill, MA, United States; ^2^School of Social and Behavioral Health Sciences, College of Public Health and Human Sciences, Oregon State University, Corvallis, OR, United States; ^3^School of Nursing, Oregon Health & Science University, Portland, OR, United States; ^4^School of Medicine, Oregon Health & Science University, Portland, OR, United States

**Keywords:** dyadic coping, dyadic illness management, depression, communication, protective buffering, active engagement

## Abstract

**Objective:**

Cancer researchers have found midlife couples to have poorer outcomes compared to older couples due to the off-time nature of the illness for them. It is unknown if young couples (aged 18–39), who are under-represented in cancer studies and overlooked for supportive programs, are at further risk. This study explored the moderating roles of survivor age and sex on the associations between active engagement and protective buffering and depressive symptoms in couples surviving cancer.

**Methods:**

The exploratory study comprised 49 couples (aged 27–58) 1–3 years post-diagnosis. Multilevel modeling was used to explore the moderating roles of survivor age and sex, controlling for interdependent data.

**Results:**

Approximately, 37% of survivors and 27% of partners met clinical criteria for further assessment of depression, with 50% of couples having at least one member meeting the criteria. Survivors and their partners did not significantly differ on depressive symptoms, active engagement, or protective buffering. Male survivors reported significantly higher levels of active engagement by their partners than female survivors and female survivors reported significantly higher levels of protective buffering by their partners than male survivors. We found some evidence to suggest that survivor age and sex may play moderating roles between active engagement and protective buffering and depressive symptoms. Older partners and female survivors appeared to experience more positive effects from engaging in positive dyadic behaviors than younger partners and male survivors.

**Conclusion:**

Findings not only confirm the important role of dyadic behaviors for couples surviving cancer together, but also the important roles of survivor age and sex may play in whether such behaviors are associated with lower levels of depressive symptoms. Future research that examines these complex associations over time and across the adult life span in diverse populations is needed.

## Introduction

It is estimated that there will be over 22 million cancer survivors in the US by 2030 (American Cancer Society, 2021). A cancer diagnosis ripples throughout the family with couples surviving cancer facing uncertainty, disruption to family goals, and high levels of fear the cancer will recur (Champion et al., 2014; Kent et al., 2016; Shapiro, 2018; Lyons et al., 2022). In addition, partners typically face the strain of providing care and support, redistribution of roles, and household tasks, but also the possibility of losing the survivor. Both survivor and partner worry about the impact of the experience on their relationship, children they may have, and the course of the family trajectory and family goals (Corney et al., 2016; Collaço et al., 2019; Gorman et al., 2020). Although not all couples living with cancer report high psychological distress or depression, and some report the experience having a positive impact on their lives and relationship (Lyons et al., 2022), research has found that some cancer survivors and partners experience high levels of anxiety and depression, even years beyond treatment (Costanzo et al., 2009; Mitchell et al., 2013; Champion et al., 2014; Kim et al., 2016; Shapiro, 2018; Lee and Lyons, 2019). A meta-analysis of predominantly mid-late life survivors reported an 11.6% (95% CI 7.7–16.2%) prevalence of depression for survivors versus 10.2% (95% CI 8.0–12.6%) for healthy controls; prevalence for survivors and their partners was not statistically significant (Mitchell et al., 2013). Much less is known about young adult couples, who are often under-represented in studies and overlooked for supportive programs (Barnett et al., 2016; Smith et al., 2016; Hydeman et al., 2019; Gorman et al., 2020), despite incidence rates of cancer increasing in this age group (Howlader et al., 2019; Scott et al., 2020).

With strong evidence of the interdependent nature of health within couples and protective role of open communication (e.g., active engagement) and collaborative behaviors (e.g., shared activities; Berg and Upchurch, 2007; Kim et al., 2016; Lyons et al., 2016b, 2018; Shaffer et al., 2016; Winters-Stone et al., 2016; Langer et al., 2017; Lyons and Lee, 2018; Acquati and Kayser, 2019; Falconier and Kuhn, 2019; Lee and Lyons, 2019; Gorman et al., 2020; Lee et al., 2020; Streck et al., 2020; Wilson et al., 2020; Hornbuckle et al., 2021; Stefanut et al., 2021), it is especially important to focus on the couple as a unit to identify at-risk couples who may be less able to support one another. Numerous dyadic theories have contributed greatly to the dyadic science of illness over the past two decades (Bodenmann, 1997; Revenson et al., 2005; Berg and Upchurch, 2007; Regan et al., 2015; Traa et al., 2015; Badr and Acitelli, 2017; Falconier and Kuhn, 2019). In particular, many of these dyadic theories highlight the importance of support and collaboration within couples through positive dyadic coping (i.e., open communication, supportive behaviors, and a sense of “we-ness”) and the need to focus on the couple as a unit (Acitelli and Badr, 2005). The current study was guided by the theory of dyadic illness management (TDIM; Lyons and Lee, 2018), which similarly moves beyond an individual perspective of illness to focus on the couple as the unit of focus. The overall goal of the theory is to optimize health within the couple by holding the health and needs of each member in balance and recognizing the heterogeneity that exists within groups and across couples.

The TDIM purports that how couples manage an illness like cancer influences the dyadic health of the couple. Integrating research on illness management, caregiving, and dyadic coping, the theory primarily focuses on the concept of dyadic management behaviors—a broad conceptualization of the collaborative verbal and non-verbal behaviors couples engage in to manage and cope with illness and survivor symptoms (i.e., communication, supportive behaviors, collaborative symptom and care management behaviors, and shared health activities; Lyons and Lee, 2018; Lyons et al., 2022). The theory also proposes the importance of shared dyadic appraisal and roles of contextual risk and protective factors at the individual (e.g., age and sex), dyadic, familial, and cultural levels in optimizing dyadic health.

Two examples of dyadic management behaviors are the relationship-focused strategies of active engagement and protective buffering (Coyne and Smith, 1991; Buunk et al., 1996). Active engagement represents open and supportive communication about the illness by one’s partner, providing opportunities to share feelings, be listened to and validated. Thus, active engagement has been considered a type of positive dyadic coping behavior (Buunk et al., 1996; Hagedoorn et al., 2000; Falconier and Kuhn, 2019), as well as a positive dyadic illness management behavior (Lyons and Lee, 2018). Alternatively, protective buffering represents the partner’s denial of or attempt to minimize illness concerns or worries, or avoidance of discussing the illness together. Thus, protective buffering has been considered a type of negative dyadic coping behavior (Buunk et al., 1996; Hagedoorn et al., 2000; Falconier and Kuhn, 2019) and negative dyadic illness management behavior (Lyons and Lee, 2018). More open and supportive communication (i.e., active engagement) has been consistently associated with more positive health in both survivors and partners (Traa et al., 2015; Lyons et al., 2016a; Acquati and Kayser, 2019; Falconier and Kuhn, 2019; Lee and Lyons, 2019; Meier et al., 2019; Streck et al., 2020; Dewan et al., 2021; Stefanut et al., 2021). However, the vast majority of studies on couples surviving cancer involves mid-late life couples and primarily breast or prostate cancer, where sex and role are often confounded. Thus, the link between active engagement and depressive symptoms by survivor age and sex is unclear, leading to one size fits all approaches (Chan et al., 2021). Furthermore, despite the term sounding positive, protective buffering has been found to be negatively associated with poor relationship outcomes, clinical-events, and depressive symptoms (Falconier et al., 2015; Lee and Lyons, 2019; Lyons et al., 2020, 2021). Given that men are sometimes more likely to “hold back” and avoid disclosures (Manne et al., 2004b, 2005, 2015), and some evidence that younger partners may engage more in protective buffering (Acquati and Kayser, 2019), exploring how these associations vary by survivor age or sex is also needed.

Transitions and experiences in life are interpreted with regard to timing in the life course (chronological, familial, and social) and the context in which they take place (Neugarten, 1979; Hareven, 1994; Berg and Upchurch, 2007). Adults do not expect to experience cancer or assume a care role for their partner in young adulthood or midlife. Although young-midlife couples may experience some commonality in the “off-time” nature of the illness, they do not share the same stage in the life span. Young adult couples (under 40) are more likely to be new to their adult roles, beginning careers and families, in shorter relationships, have less experience with collaborative coping skills and health behaviors, and are more susceptible to contextual factors, such as financial strain and illness (Berg and Upchurch, 2007; American Psychological Association, 2019; Hydeman et al., 2019). The long-term effects of cancer and implications for the young adult couple (e.g., fertility, disruptions to career and family goals, and changes in relationships) may be especially challenging (Barnett et al., 2016; Corney et al., 2016; Collaço et al., 2019; Hydeman et al., 2019).

Researchers have consistently found younger survivors and younger couples to have more negative outcomes compared with older survivors and older couples (Harden et al., 2006; Champion et al., 2014; Borstelmann et al., 2015; Rottmann et al., 2016; Lee and Lyons, 2019), but broad age ranges in studies make it difficult to purposely examine age risk when younger couples are often poorly represented. A notable exception includes a cross-sectional study of couples living with breast cancer that compared 35 young couples (aged 45 or younger) to 51 midlife couples (aged 46–66) within 3 months of diagnosis that found younger survivors had worse physical and mental health, greater negative impact of cancer and less social support than midlife survivors. Similarly, younger partners had worse mental health than midlife survivors (Acquati and Kayser, 2019). No differences in dyadic coping were found between age-groups for survivors, but younger partners had more negative dyadic coping than midlife partners (Acquati and Kayser, 2019). It is unknown if the theoretically purported benefits of positive dyadic behaviors, such as active engagement and low levels of protective buffering on depressive symptoms, vary by survivor age.

Similarly, cancer research has predominantly focused on breast and prostate cancer, where sex and role are often confounded (e.g., all survivors in the study are female), limiting the ability to fully understand the role of survivor sex in how couples experience and navigate cancer. Evidence suggests females, regardless of role, experience more negative outcomes than males (Hagedoorn et al., 2008; Falconier and Kuhn, 2019). Although female survivors have been found to engage in more positive dyadic behaviors, such as open communication and support (Acquati and Kayser, 2019), and to be more likely than males to collaborate and define themselves relationally (Kiecolt-Glaser and Newton, 2001; Berg and Upchurch, 2007), they may also be more vulnerable when such collaboration is absent (Kiecolt-Glaser and Newton, 2001; Berg and Upchurch, 2007; Lyons et al., 2018). Given the importance placed on collaborative dyadic behaviors (particularly behaviors, such as active engagement) as protective for couples experiencing cancer, more deliberate research on the role of survivor sex in couples across the life span is needed to address this gap.

Thus, the current study builds upon previous research and is guided by the TDIM to explore the moderating roles of survivor age and sex (i.e., individual contextual factors) on the associations between active engagement and protective buffering (i.e., dyadic illness management behaviors) and depressive symptoms of survivor and partner (i.e., dyadic health) in young-midlife couples 1 to 3 years after diagnosis.

## Materials and Methods

The current exploratory study recruited couples through the Oregon State Cancer Registry *via* targeted mailings. Per the cancer registry’s protocol, letters describing the study were mailed by registry staff to survivors meeting initial eligibility criteria (i.e., diagnosis data, age, and zip code to optimize representation of both rural and urban-dwelling couples). A total of 700 letters were mailed with equal numbers sent to young survivors (aged 21–39 at diagnosis) and midlife survivors (aged 40–56 at diagnosis). Additionally, recruitment flyers were posted in an oncology clinic at Oregon Health and Science University (OHSU). All interested couples were screened for eligibility by research staff at OHSU. Eligibility criteria included that (1) the survivor had a primary diagnosis of invasive cancer in the preceding 18–36 months, (2) couples were co-residing at the time of diagnosis and recruitment, (3) couples were aged 21 to 56 years at diagnosis, (4) couples had the ability to read English, (5) couples had access to a telephone, and (6) couples were resident in Oregon. We selected the upper bound of 56 years of age to minimize inclusion of couples considering retirement at time of diagnosis. Couples were not required to be married and couples of any sexual orientation were eligible to participate.

A total of 160 survivors expressed interest in the study (158 from the targeted registry mailings—23% response rate—and two from the fliers posted in the oncology clinic). Thirty-three survivors (21%) were lost to follow-up and could not be reached for a screening phone call, even after several attempts. A total of 77 couples (48%), who were screened by phone, were eligible. The remaining 50 survivors (31%) were screened as ineligible because they did not have a partner (36%), were older than 56 at diagnosis (40%), did not meet diagnosis criteria/reason unknown (10%), could not read English (4%), the survivor had died (6%), or declined to participate (4%). The 77 eligible couples were mailed a packet containing separate surveys for survivor and partner and separate consent forms. Couples were asked to complete surveys independently and return them with signed consent forms in the provided stamped-addressed envelopes. A total of 49 couples (64%) returned surveys and signed consent forms for both survivor and partner. The study was approved by the Institutional Review Board at Oregon Health and Science University (e#15498).

### Measures

All sociodemographic information and measures for both members of the couple were obtained through their respective mail surveys.

#### Depressive Symptoms

Depressive symptoms were measured with the Center for Epidemiological Studies Depression scale that has good internal consistency, sensitivity, specificity, and validity (Radloff, 1977; Beekman et al., 1997), including in couples with cancer (Lyons et al., 2014). Survivors and partners responded to 20 statements using a 0 (rarely or none) to 3 (most or all) scale (e.g., “I was bothered by things that do not usually bother me,” “I felt depressed,” and “I did not feel like eating/appetite was poor”). Scores were summed with higher scores indicating greater depressive symptomatology. A score of 16 or above indicates likely depression and the need for further assessment (Radloff, 1977). More recent research suggests a clinical cut-off score of 20 or above may have a more adequate trade-off of sensitivity and specificity for depression (Vilagut et al., 2016). Cronbach’s alpha in the current study was 0.91 for survivors and 0.92 for partners.

#### Dyadic Illness Management Behaviors

Dyadic illness management behaviors are operationalized in two ways in the current study—active engagement and protective buffering. Active engagement and protective buffering were measured using the two subscales of the Dyadic Coping measure (Buunk et al., 1996; Hagedoorn et al., 2000). The active engagement subscale has five items that assess the extent to which the survivor and partner view each other’s active involvement and support (e.g., “my partner tries to discuss cancer with me openly,” “when something bothers me, my partner tries to discuss it with me,” and “my partner is full of understanding towards me”). Participants respond to the five items using a Likert scale from 1 (never) to 5 (very often). Higher scores indicate higher levels of perceived active engagement by one’s partner. The scale has exhibited strong internal consistency in studies of couples with cancer (Hagedoorn et al., 2000; Hinnen et al., 2007), including the current study (Cronbach’s alpha for survivor = 0.89; Cronbach’s alpha for partner = 0.81). The protective buffering subscale consists of six items that assess the extent to which the survivor and partner view each other’s use of hiding concerns and denying worries (e.g., “my partner tries to hide his or her worries about me,” “my partner just waves my worries aside,” and “my partner tries to act like nothing is the matter”). Participants respond to six items using a Likert scale from 1 (never) to 5 (very often). Higher scores indicate higher levels of perceived protective buffering by one’s partner. The scale has exhibited good internal consistency in studies of couples with cancer (Hinnen et al., 2007), including the current study (Cronbach’s alpha for survivor = 0.77; Cronbach’s alpha for partner = 0.65).

### Analysis Plan

Descriptive statistics were used to characterize the sample (SPSS v26; IBM Corporation, Armonk, NY). Paired samples t-tests were used to compare survivor and partner depressive symptoms, active engagement and protective buffering due to the interdependent nature of the data. Multilevel modeling (Hierarchical Linear Modeling v8; Skokie, IL) was used to explore the moderating roles of survivor age and sex on the associations between active engagement and protective buffering and depressive symptoms at the level of the couple to control for interdependencies between survivor and partner data (Garcia et al., 2015). HLM uses full information maximum likelihood estimation, which estimates parameter values based on all existing data available to obtain unbiased estimates.

Two models were run to explore the moderating role of survivor age (as a continuous variable) on the association between each dyadic behavior (i.e., active engagement and protective buffering) and depressive symptoms. Each model included a moderated actor term that represented the interaction between survivor age and survivor report of the dyadic behavior (i.e., active engagement and protective buffering) and a moderated partner term that represented the interaction between survivor age and partner report of the dyadic behavior (i.e., active engagement and protective buffering; Garcia et al., 2015). A significant interaction effect was deemed evidence of moderation. Due to the small sample size, effect sizes $r=\sqrt{\frac{t^{2}}{\left(t^{2}+df\right)}}$ were calculated and reported. Only results with medium (*r* = 0.30) or large (*r* = 0.50) effects were interpreted. Figures depict each variable’s high (1 *SD* above the mean) and low (1 *SD* below the mean) values. A similar procedure was used to examine the moderating role of survivor sex on the association between each dyadic behavior (i.e., active engagement and protective buffering) and depressive symptoms. Variables were centered prior to creating interaction terms except for survivor sex, which was coded as 0 (male) and 1 (female).

Survivor age was treated as a continuous variable in moderation analyses due to significant disadvantages of dichotomizing data including significant loss of information, variability, statistical power (especially in small samples), and higher risk of false positive results (Altman and Royston, 2006). Thus, the role of age in moderation analyses is interpreted as the role of increasing/decreasing age (or being older or younger) across young-mid adulthood.

## Results

### Sample Characteristics

Table 1 displays the sociodemographic and background characteristics for the sample of 49 couples. Survivors and partners were, on average, 43.5 (*SD* = 9.0) and 43.9 (*SD* = 9.7) years old, respectively, with 43% of the sample between the ages of 27 and 40. Survivors were predominantly female (69%), white (90%), non-Hispanic (88%), employed (61%), and had completed college. Average time since diagnosis was 2.26 (*SD* = 0.60) years. Partners were predominantly male (67%), white (82%), non-Hispanic (88%), employed (76%), and completed college (59%). Almost half of couples lived in designated rural areas. The small number of Hispanic couples (12%) was primarily rural dwelling and under 40 years of age. Breast cancer was the most common diagnosis for survivors (20%), followed by cervical/ovarian (13%), colon (10%), and renal (10%).

**Table 1:** Demographics and characteristics of survivors and partners (*n* = 49 couples).

Participant characteristics	Survivors M ± SD or *n* (%)	Partner M ± SD or *n* (%)	*t* statistic	Cohen’s *d*	Correlation
Age (years)	43.5 ± 9.0	43.9 ± 9.7	−0.43		0.79^***^
Sex (% female)	34 (69%)	16 (33%)			
Race (% white)	44 (90%)	40 (82%)			
Ethnicity (% Hispanic)	6 (12%)	6 (12%)			
Education (% completed college)	36 (74%)	29 (59%)			
Employment (% employed)	30 (61%)	37 (76%)			
Residence (% rural location)	22 (45%)	–			
Length of co-residence (years)	16.6 ± 9.9	–			
Years since diagnosis	2.2 ± 0.6	–			
Depressive symptoms (0–60)	15.5 ± 11.3	12.6 ± 10.2	1.49	0.22	0.22
Active engagement (0–20)	13.4 ± 4.7	13.2 ± 3.8	0.26	0.04	0.30^*^
Protective buffering (0–24)	9.2 ± 4.9	7.5 ± 3.7	1.91	0.29	0.02

Differences between survivors and partners were examined using paired *t*-tests. Correlations represent paired samples correlations between survivors and partners.

* *p* < 0.05 and

*** *p* < 0.001.

### Differences in Depressive Symptoms, Active Engagement, and Protective Buffering by Role, Survivor Age Group, and Survivor Sex

Table 1 includes comparisons by role (i.e., survivor versus partner) in depressive symptoms, active engagement, and protective buffering. On average, survivor depressive symptoms were 15.5 (*SD* = 11.3) with 37% of survivors at or above a score of 16 (clinical cut-off for further assessment). Partner depressive symptoms were, on average, 12.6 (*SD* = 10.2) with 27% of partners at or above a score of 16. Half of couples in the sample had at least one member meeting the clinical cut-off (17% of couples had both members). There were no significant differences found between survivors and partners on depressive symptoms, or in perceived active engagement and protective buffering. Depressive symptoms between survivors and partners were correlated at 0.22, indicating some covariation. Reports of active engagement were similarly correlated within couples. However, survivor and partner reports of protective buffering showed little to no correlation.

Table 2 includes comparisons by survivor age group (aged <40 versus aged 40 and older) in depressive symptoms, active engagement, and protective buffering. There were no significant differences in survivor or partner depressive symptoms, active engagement, or protective buffering behaviors by age group. Although not statistically significant, medium effects suggest younger survivors were more likely to report higher levels of active engagement by their partner (Cohen’s *d* = 0.50) than midlife survivors; younger partners were more likely to report higher levels of active engagement by their survivor (Cohen’s *d* = 0.55).

**Table 2:** Comparison of depressive symptoms, active engagement, and protective buffering by survivor age group (*n* = 49 survivors).

Variable	Young SVRs (<40 years old) M ± SD	Midlife SVRs (> 40 years old) M ± SD	*t* statistic	Cohen’s *d*
SVR depressive symptoms	13.4 ± 9.1	16.6 ± 12.2	−1.35	−0.29
Partner depressive symptoms	9.7 ± 10.4	13.9 ± 9.9	−1.35	−0.42
SVR active engagement	15.0 ± 3.6	12.7 ± 5.1	1.68	0.50
Partner active engagement	14.5 ± 3.8	12.5 ± 3.6	1.83	0.55
SVR protective buffering	8.7 ± 4.8	9.7 ± 5.0	−0.64	−0.19
Partner protective buffering	7.4 ± 4.1	7.2 ± 3.8	0.14	0.04

SVR, survivor.

Table 3 includes comparisons by survivor sex in depressive symptoms, active engagement, and protective buffering. There were no significant differences in depressive symptoms by survivor sex for survivors or partners. Significant sex differences were found for survivor-reported active engagement and survivor-reported protective buffering. Male survivors (*p* < 0.05; Cohen’s *d* = 0.57) reported significantly higher active engagement by their partner than female survivors. Female survivors (*p* < 0.05; Cohen’s *d* = −0.74) reported significantly higher protective buffering by their partner than male survivors. No significant differences were found for partners.

**Table 3:** Comparison of depressive symptoms, active engagement, and protective buffering by survivor sex (*n* = 49 survivors).

Variable	Female SVRs M ± SD	Male SVRs M ± SD	*t* statistic	Cohen’s *d*
SVR depressive symptoms	16.3 ± 11.7	13.3 ± 9.8	−0.86	−0.27
Partner depressive symptoms	12.1 ± 7.8	13.4 ± 14.6	0.313	0.13
SVR active engagement	12.8 ± 5.3	14.4 ± 2.6	2.37^*^	0.57
Partner active engagement	12.9 ± 3.6	13.9 ± 4.3	0.85	0.27
SVR protective buffering	10.4 ± 5.1	6.9 ± 3.6	−2.37^*^	−0.74
Partner protective buffering	7.5 ± 3.6	6.8 ± 4.3	−0.60	−0.19

SVR, survivor.

* *p* < 0.05.

### Moderating Role of Survivor Age on the Associations Between Active Engagement and Protective Buffering and Depressive Symptoms

**Figure 1:**
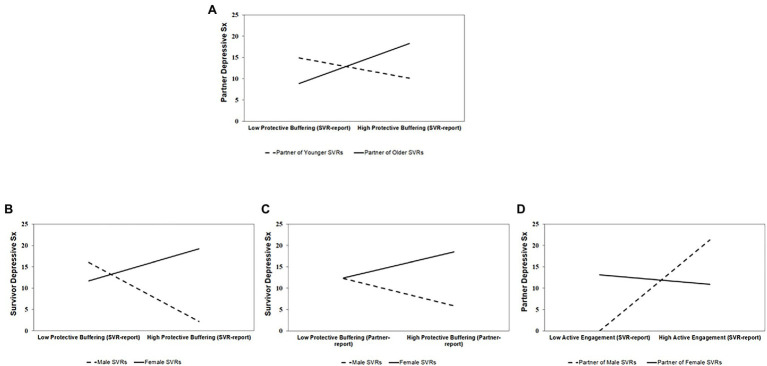
The moderating effects of survivor age and sex on the associations between active engagement and protective buffering and depressive symptoms. Panel **(A)** shows the moderating role of survivor age on the association between survivor-reported protective buffering on partner depressive symptoms. Panels **(B–D)** show the moderating role of survivor sex on the association of survivor-reported protective buffering on survivor depressive symptoms, partner-reported protective buffering on survivor depressive symptoms, and survivor-reported active engagement on partner depressive symptoms, respectively. High and low values represent 1 SD above and below the mean on age, active engagement, and protective buffering. Sex was coded 0 (males) and 1 (females).

Table 4 includes the results of the moderation analysis using age as a continuous variable. One significant interaction was found. First, we found evidence of a moderated partner effect in that survivor age significantly moderated the association between the survivor’s perception of protective buffering (in their partner) and partner depressive symptoms [*p* < 0.5; ES (*r*) = 0.32]. Older partners, whose survivors perceived them to engage in lower protective buffering, reported lower depressive symptoms. In contrast, younger partners, whose survivor perceived them to engage in lower protective buffering, reported higher depressive symptoms (Figure 1A). No significant moderation effects were found for survivor depressive symptoms.

**Table 4:** Moderating role of survivor age on associations between active engagement and protective buffering and depressive symptoms (*n* = 49 couples).

Variables	Depressive symptoms			
	SVRs		Partners	
	B (SE)	ES (r)	B (SE)	ES (r)
**Active engagement**
SVR age	0.18 (0.19)	0.14	0.11 (0.18)	0.09
SVR-reported active engagement	−0.28 (0.37)	0.12	0.21 (0.33)	0.10
Partner-reported active engagement	0.18 (0.19)	0.14	−0.08 (0.42)	0.03
SVR age^*^SVR-reported active engagement	−0.03 (0.04)	0.11	−0.02 (0.04)	0.08
SVR age^*^Partner-reported active engagement	0.05 (0.05)	0.17	−0.07 (0.04)	0.22
**Protective buffering**
SVR age	0.20 (0.17)	0.19	0.06 (0.16)	0.06
SVR-reported protective buffering	0.56 (0.32)	0.27	0.24 (0.30)	0.02
Partner-reported protective buffering	0.34 (0.45)	0.12	0.74 (0.42)	0.28
SVR age^*^SVR-reported protective buffering	0.02 (0.04)	0.09	0.08 (0.04)^*^	0.32
SVR age^*^Partner-reported protective buffering	0.01 (0.05)	0.02	0.07 (0.04)	0.25

B, unstandardized coefficient; SVR, survivor. Survivor age was included as a continuous variable. Both survivor age and both dyadic management behavior variables were centered to create interaction terms. Higher scores on active engagement and protective buffering indicate higher levels of each behavior. Effect size $r=\sqrt{\frac{t^{2}}{\left((,t^{2}+df,)\right)}}$ .

* *p* < 0.05.

### Moderating Role of Survivor Sex on the Associations Between Active Engagement and Protective Buffering and Depressive Symptoms

Table 5 includes the results of the analysis exploring survivor sex as a moderator. Three significant interactions were found. First, we found evidence of a moderated actor effect in that survivor sex significantly moderated the association between survivor’s report of protective buffering (by their partner) on survivor depressive symptoms [*p* < 0.05; ES (*r*) = 0.39]. Female survivors, who reported fewer depressive symptoms, were significantly more likely to report their partners engaging in low levels of protective buffering. Male survivors, who reported fewer depressive symptoms, were significantly more likely to report their partner engaging in high levels of protective buffering (Figure 1B).

**Table 5:** Moderating role of survivor sex on associations between active engagement and protective buffering and depressive symptoms (*n* = 49 couples).

**Variables**	**Depressive symptoms**			
	SVRs		Partners	
	B (SE)	ES (r)	B (SE)	ES (r)
**Active engagement**
SVR sex	6.31 (4.12)	0.05	3.75 (3.62)	0.16
SVR-reported active engagement	1.65 (1.20)	0.21	2.78 (1.01)^**^	0.39
Partner-reported active engagement	0.23 (0.72)	0.05	−0.44 (0.62)	0.11
SVR sex^*^SVR-reported active engagement	−2.07 (1.26)	0.25	−3.01 (1.07)^**^	0.40
SVR sex^*^Partner-reported active engagement	−0.25 (0.91)	0.04	0.60 (0.79)	0.01
**Protective buffering**
SVR sex	6.36 (3.70)	0.27	−2.13 (3.88)	0.09
SVR-reported protective buffering	−1.40 (0.75)	0.29	0.24 (0.83)	0.05
Partner-reported protective buffering	−0.83 (0.60)	0.22	0.92 (0.71)	0.21
SVR sex^*^SVR-reported protective buffering	2.16 (0.83)^*^	0.39	−0.08 (0.91)	0.01
SVR sex^*^Partner-reported protective buffering	1.63 (0.80)^*^	0.31	−0.86 (0.91)	0.15

B, unstandardized coefficient; SVR, survivor. Survivor sex was coded 0 (male) and 1 (female). Both dyadic management behavior variables were centered to create interaction terms. Higher scores on active engagement and protective buffering indicate higher levels of each behavior. Effect size $r=\sqrt{\frac{t^{2}}{\left(t^{2}+df\right)}}$ .

* *p* < 0.05.

** *p* < 0.01.

Second, we found evidence of a moderated partner effect in that survivor sex significantly moderated the association between partner’s report of protective buffering (by the survivor) on survivor depressive symptoms [*p* < 0.05; ES (*r*) = 0.31]. Female survivors, who reported fewer depressive symptoms, were significantly more likely to have partners, who perceived them (the survivor) as engaging less in protective buffering. Male survivors, who reported fewer depressive symptoms, were significantly more likely to have partners, who perceived them (the survivor) as engaging more in protective buffering (Figure 1C).

Third, we found evidence of another moderated partner effect in that survivor sex significantly moderated the association between survivor’s report of active engagement (by their partner) on partner depressive symptoms [*p* < 0.01; ES (*r*) = 0.40]. The depressive symptoms reported by partners of female survivors (almost all men) had little to no association with how the female survivor perceived the active engagement of the partner. In contrast, partners of male survivors (almost all women) had more depressive symptoms when the male survivor reported them as engaging in more active engagement (Figure 1D).

## Discussion

This study set out to explore the moderating roles of survivor age and sex on the associations between active engagement and protective buffering and depressive symptoms among young-midlife couples 1 to 3 years after diagnosis. Although this study was limited by sample size, there are several noteworthy findings to inform future work. First, we found that over a third of survivors and a quarter of partners experienced high enough depressive symptomatology 1 to 3 years post-diagnosis to require further clinical assessment. We found no significant differences in level of depressive symptoms between survivors and partners or by survivor age group or sex. Within couples, we found that half of couples had at least one member (17% had both members) scoring above the clinical cut-off for further assessment. Second, although active engagement and protective buffering behaviors did not differ significantly between survivors and partners, we did find group differences by survivor sex. Male survivors reported significantly higher levels of active engagement by their partners than female survivors and female survivors reported significantly higher levels of protective buffering by their partners than male survivors. Finally, we found some evidence to suggest that survivor age and sex may play moderating roles between these behaviors and depressive symptoms. Older partners and female survivors appeared to experience more positive effects than younger partners and male survivors.

Consistent with previous research (Mitchell et al., 2013; Champion et al., 2014; Shapiro, 2018), this study found that some couples surviving cancer beyond the first year still experience depressive symptoms. In contrast to a study of 1,127 couples 3–8 years post-diagnosis that reported 18–27% of survivors with likely clinical depression requiring further assessment, our study (using similar clinical cut-offs) found 37% of survivors and 27% of partners requiring further assessment (Champion et al., 2014). Even using a more conservative cut-off of 20 that has been found to have a more adequate sensitivity-specificity balance for depression (Vilagut et al., 2016), our sample had 29% of survivors and 23% of partners meeting criteria. Moreover, 50% of our couples had one or both members meeting criteria. Unlike recent research on couples living with breast cancer, we did not find significant age-group differences (Acquati and Kayser, 2019) or survivor sex differences in depressive symptoms. As so little couple research in cancer has focused on cancers involving both sexes in young to mid-adulthood, replication of findings is needed before drawing strong conclusions about the lack of sex differences, though we acknowledge that 69% of survivors in our sample were female.

Despite the growing body of literature supporting the protective roles of open communication, supportive behaviors, and shared collaborative activities on the interdependent health of the dyad (Berg and Upchurch, 2007; Berg et al., 2008; Falconier et al., 2015; Regan et al., 2015; Traa et al., 2015; Shaffer et al., 2016; Lyons and Lee, 2018; Streck et al., 2020; Stefanut et al., 2021), few studies have explicitly examined whether such beneficial effects vary by survivor age or sex. Our findings suggest that the beneficial effects of such behaviors may not be universal.

Specifically, older partners (i.e., as age increased) experienced lower depressive symptoms when the survivor reported them as engaging in less protective buffering. Thus, partners, in our sample, more likely to be older (i.e., in midlife) than younger, benefitted from low levels of protective buffering. Yet, younger partners did not appear to benefit similarly in the current sample. Younger couples tend to be newer to their relationships with one another (as was true in our study) and may be less experienced in these types of positive communication skills and supportive behaviors than older couples (Berg and Upchurch, 2007), with recent evidence that younger partners engage in significantly higher levels of negative dyadic behaviors than midlife partners (Acquati and Kayser, 2019). The off-time nature of the cancer experience and unexpected role of care partner may be particularly challenging for younger-aged partners, who may be unsure of how to emotionally support the survivor over time and the appropriateness of balancing their own needs.

Relatedly, the social cognitive processing theory (Lepore and Revenson, 2007) purports that our psychological health is influenced by our ability to process and discuss traumatic events, such as a cancer diagnosis, with those who are closest to us. When attempts to discuss or communicate openly about the experience with one’s partner or family member is perceived to be met with unsupportive responses or social constraints, the person attempting to share can be hindered in their ability to cognitively process the experience, leading to intrusive thoughts and cognitive avoidance, and ultimately higher depressive symptoms (Lepore and Revenson, 2007; Cohee et al., 2017). Several items on the protective buffering measure are similar to those on measures of social constraint by one’s partner (e.g., “my partner tries to hide worries about me,” “my partner tries to act as if nothing is the matter,” “my partner just waves my worries aside,” and “my partner does everything to prevent me from thinking about my cancer”). Thus, when young survivors in the current sample reported high levels of what could be considered social constraint behaviors by their partners, partners reported low levels of depressive symptoms. It is possible that younger partners are experiencing some benefit from not engaging in open communication and discussion about the cancer experience either because they see their supportive role as one of protection through denial and distraction (Manne et al., 2004a, 2005, 2015; Lepore and Revenson, 2007), because they lack skills and confidence to openly communicate, or such supportive and open communication about their partner’s cancer may be too emotionally draining for them (Ernst et al., 2017; Crangle et al., 2020; Reblin et al., 2020). It is also possible they perceived social constraints from the survivor. All of which highlight the complexity of communication and support within couples experiencing illness and need for couple-based interventions to facilitate these skills of sharing and reciprocal disclosure, particularly for those in care partner and supportive roles, who often feel guilty about expressing their own needs and challenges (Spillers et al., 2008; Yeung et al., 2018).

However, given the cross-sectional nature of the study, it is also possible that younger partners with high levels of depressive symptoms were more likely to be perceived by the younger survivor as engaging in less protective buffering. Younger couples may not want or be unsure of how to openly discuss due to their earlier stage in life and early stage of the relationship, and may be more prone to want to move on and avoid discussion or believe this is the positive thing to do (Pistrang and Barker, 2005). Clearly, much more research is needed to untangle these effects and how they unfold for the younger couple over time.

Similarly, we found differential effects by survivor sex. Female survivors, who reported few depressive symptoms, were either significantly more likely to report their partners engaging in lower levels of protective buffering or were more likely to have partners who perceived them (the survivor) as engaging less in protective buffering. The same beneficial effect was not observed for male survivors. This raises the potential of how male survivors are interpreting the meaning and value of protective buffering behaviors (by themselves and their partners) and whether we are detecting engendered effects about the relational nature of women’s identities versus the social expectations of masculinity that may drive patterns of distraction and denial/avoidance by men in illness contexts (Manne et al., 2005, 2015; Pistrang and Barker, 2005; Badr and Carmack Taylor, 2006; Mahalik and Dagirmanjian, 2019). Men, across races, ethnicities, and the adult life span, have been found to seek help for mental health and depression less than women (Addis and Mahalik, 2003). Indeed, recent research suggested that husbands of women with breast cancer, who highly endorsed masculine strength, experienced significantly higher levels of guilt when they did seek help and those husbands who engaged in protective buffering experienced significantly less guilt (Yeung et al., 2018).

Finally, partners of male survivors (all women but one) reported higher levels of depressive symptoms when the survivor perceived them to be engaged in high levels of active engagement. It is unclear if this indicates that partners experiencing higher levels of depressive symptoms were prompted to engage in more open communication to deal with what they were experiencing and as a way to process and seek support rather than the engagement in open communication leading to depressive symptoms. Though social cognitive processing theory would suggest that if those active engagement behaviors, by the predominantly female partners in the study, were met with social constraint, they could lead to poor mental health over time (Lepore and Revenson, 2007).

It is unfortunately not possible to untangle the full story behind the effects observed in the current study, nor tease apart what may well be an intersection of age and sex in how styles of communication and ability to openly communicate, receive the communication and reciprocally disclose are interpreted and used. Social constraints arise not only from the environment and social context present, but are also strongly driven by the interpretation of the person disclosing (Lepore and Revenson, 2007), which may not be readily understood by the person hearing that disclosure. Although much of the work around couple communication in cancer and other illnesses has focused on the survivor’s disclosures and the supportive/unsupportive behaviors and responses of their partner, a more balanced approach to the transactions within couples and partner’s disclosures may lead to a more shared understanding of the positive ways to communicate and support, promote reciprocal disclosures, empathetic listening, and perspective-taking by both. In addition to the potential roles of age and sex in dyadic behaviors, the family care literature highlights some of the misplaced assumptions and guilt care partners can feel in voicing their own needs and challenges, particularly for male and younger partners (Spillers et al., 2008; Yeung et al., 2018). This guilt and perceived role expectations to remain positive and focus solely on the needs of their partner with illness may hinder the couple from achieving healthy, long-term communication, and mutually supportive skills and strategies. Research has consistently found that care partners also experience negative outcomes and poor health as in the current study (Kent et al., 2016; Kim et al., 2016; Shaffer et al., 2016), sometimes at significantly higher levels than the person with illness (Lee and Lyons, 2019).

### Limitations

There are several important limitations to the current work. First, the sample is small and cross-sectional. This not only prevents us from drawing any conclusions about directionality of associations (though theory strongly guided our research questions), but we are underpowered to adequately test moderation and draw strong conclusions from our findings. We tried to ameliorate the small sample by focusing only on those results with medium-large effect sizes and using age as a continuous variable in the moderation analyses (Altman and Royston, 2006), but call for replication of our results in larger, more diverse samples. Second, our homogeneous sample also lacked racial diversity and included only one same-sex couple preventing us from examining these associations within groups of couples. Thus, it is possible that in larger, more diverse samples or samples focused solely on couples under-represented in couple research, we may uncover other contexts where the benefits of dyadic behaviors are not present or are unclear. Finally, we did not limit our sample to a certain type of cancer or group of cancers as we deliberately wanted to explore survivor sex separate from role (i.e., survivor versus partner). We acknowledge the heterogeneity that this introduces, but given that our guiding theory purports to be relevant for most illness contexts, we believe this inclusive criterion has provided salient information. Finally, we did not include a measure of collaborative illness management in the study so cannot compare the more non-verbal ways couples collaborate and support one another in the context of illness.

### Strengths and Implications

Despite these limitations, this population-based study contributes to the field of dyadic science in cancer in several ways. First, our results question the universal benefits of more open communication and the potentially salient roles of survivor age and sex. It is our hope that these results will guide future directions for more deliberate research to examine these processes within larger samples over time in more nuanced ways. Moreover, to truly examine the role of age in dyadic processes and behaviors, samples should purposely include couples across the entire adult life span (Acquati and Kayser, 2019). Second, we believe our inclusion of more than one cancer increases the generalizability of our findings and prompts further work to tease apart the intersectionality of sex, age, and role. Third, our purposeful focus and recruitment of couples under the age of 40 1–3 years post-diagnosis from both rural and urban areas adds to the emerging body of research on the ongoing challenges and experiences of these overlooked couples. Finally, our findings highlight the ongoing emotional strain experienced by some young and midlife couples surviving cancer after the first year of diagnosis.

We see several implications from this work. Examining the roles of sex and age within diverse groups of couples is needed to understand how dyadic processes hold up in different cultures and contexts across the life span. Moreover, the role of family around the couple is often neglected from the dyadic science of illness, yet the family context and family relationships can play important (albeit different) roles for couples across the life span and in different cultures (Carter et al., 2010; Jeong et al., 2018; Bonds Johnson et al., 2021). We have found familial support to be an important factor for dyadic outcomes in much of our research (Lyons and Lee, 2018; Lyons et al., 2021, 2022). Combining dyadic theories of health and illness with more specific theories of social cognitive processing and communication may lead to more balanced and nuanced ways to design couple-based interventions that not only facilitate communication skills and non-verbal collaboration, but also acknowledge the challenges for care partners, the readiness to share and listen within the couple, and times when other supporters beyond the couple may be beneficial. Furthermore, supportive and open communication is just one way that couples collaborate and manage cancer together. The TDIM purports that dyadic illness management behaviors comprise of not just open communication, but also supportive behaviors to survivor and care partner, shared health behaviors, and collaborative illness and care management behaviors (Lyons and Lee, 2018; Lyons et al., 2022). Thus, multicomponent couple-based interventions and approaches may be optimal and provide maximum tailoring to the specific needs and challenges of couples.

Clearly, one size does not fit all couples and challenges around communication and support may change with the cancer trajectory, stage of cancer, place in the life span, stage of the relationship, the sex of both survivor and partner, and their role in the relationship. Younger couples may be particularly in need of interventions to learn to cope with stress compared to older couples who may have already weathered challenges together. Similarly, some men (particularly in partner roles) may benefit from more nuanced approaches to open communication that acknowledge the role of more traditional masculine identities and potential lack of skill and experience with disclosure and open communication. Research that delves deeper into the ways couples communicate and collaborate and identifies couples who are most vulnerable and unable to support one another is needed. Recent longitudinal work examining communication and relationship outcomes using ecological momentary assessment is a noteworthy example (Langer et al., 2018). The dyadic science of illness and health has led to important and relevant theories and knowledge and directly informed effective couple-based interventions (Li and Loke, 2014; Winters-Stone et al., 2016; Langer et al., 2018; Hornbuckle et al., 2021; Reese et al., 2021). This study not only supports that work but prompts further exploration of when, why, and for whom these processes do not lead to beneficial outcomes.

## Data Availability Statement

The raw data supporting the conclusions of this article will be made available by the authors, without undue reservation.

## Ethics Statement

The studies involving human participants were reviewed and approved by the Institutional Review Board at Oregon Health and Science University (e#15498). The patients/participants provided their written informed consent to participate in this study.

## Author Contributions

KL and BH-L contributed to the conceptualization and design of the study. JG contributed to the conceptualization of the study. BL contributed to the data management and preliminary analysis. GD contributed to the preliminary analysis. KL wrote the first draft of the manuscript. KL and JG wrote sections of the manuscript. All authors contributed to the manuscript revision, read, and approved the submitted version.

## Funding

This work was generously supported by the Betty Gray Foundation, Oregon.

## Conflict of Interest

The authors declare that the research was conducted in the absence of any commercial or financial relationships that could be construed as a potential conflict of interest.

## Publisher’s Note

All claims expressed in this article are solely those of the authors and do not necessarily represent those of their affiliated organizations, or those of the publisher, the editors and the reviewers. Any product that may be evaluated in this article, or claim that may be made by its manufacturer, is not guaranteed or endorsed by the publisher.
